# Derivation of metabolic point of departure using high-throughput in vitro metabolomics: investigating the importance of sampling time points on benchmark concentration values in the HepaRG cell line

**DOI:** 10.1007/s00204-022-03439-3

**Published:** 2023-01-22

**Authors:** Julia M. Malinowska, Taina Palosaari, Jukka Sund, Donatella Carpi, Ralf J. M. Weber, Gavin R. Lloyd, Maurice Whelan, Mark R. Viant

**Affiliations:** 1 School of Biosciences, University of Birmingham, Birmingham, B15 2TT UK; 2 European Commission, Joint Research Centre (JRC), Ispra, Italy; 3 Phenome Centre Birmingham, University of Birmingham, Birmingham, B15 2TT UK

**Keywords:** In vitro metabolomics, HepaRG, Direct infusion mass spectrometry, Benchmark concentration analysis, Point-of-departure, Chemical risk assessment

## Abstract

Amongst omics technologies, metabolomics should have particular value in regulatory toxicology as the measurement of the molecular phenotype is the closest to traditional apical endpoints, whilst offering mechanistic insights into the biological perturbations. Despite this, the application of untargeted metabolomics for point-of-departure (POD) derivation via benchmark concentration (BMC) modelling is still a relatively unexplored area. In this study, a high-throughput workflow was applied to derive PODs associated with a chemical exposure by measuring the intracellular metabolome of the HepaRG cell line following treatment with one of four chemicals (aflatoxin B_1_, benzo[a]pyrene, cyclosporin A, or rotenone), each at seven concentrations (aflatoxin B_1_, benzo[a]pyrene, cyclosporin A: from 0.2048 μM to 50 μM; rotenone: from 0.04096 to 10 μM) and five sampling time points (2, 6, 12, 24 and 48 h). The study explored three approaches to derive PODs using benchmark concentration modelling applied to single features in the metabolomics datasets or annotated metabolites or lipids: (1) the 1st rank-ordered unannotated feature, (2) the 1st rank-ordered putatively annotated feature (using a recently developed HepaRG-specific library of polar metabolites and lipids), and (3) 25th rank-ordered feature, demonstrating that for three out of four chemical datasets all of these approaches led to relatively consistent BMC values, varying less than tenfold across the methods. In addition, using the 1st rank-ordered unannotated feature it was possible to investigate temporal trends in the datasets, which were shown to be chemical specific. Furthermore, a possible integration of metabolomics-driven POD derivation with the liver steatosis adverse outcome pathway (AOP) was demonstrated. The study highlights that advances in technologies enable application of in vitro metabolomics at scale; however, greater confidence in metabolite identification is required to ensure PODs are mechanistically anchored.

**Supplementary Information:**

The online version contains supplementary material available at 10.1007/s00204-022-03439-3.

## Introduction

New Approach Methodologies (NAMs) are increasingly recognised as important tools in chemical safety assessment for advancing the paradigm shift in toxicity testing without the use of vertebrate animals. Amongst them, omics technologies offer the possibility of comprehensively measuring gene expression (transcriptomics), protein abundance (proteomics) or metabolite levels (metabolomics) in a biological system exposed to a chemical. Metabolomics studies—measuring biochemicals with molecular weight < 1.5 kDa—allow the downstream metabolic phenotype of cells, tissues, or whole organisms to be established, which is arguably the closest phenotype provided by all omics technologies to traditional apical endpoints used in toxicology. In recent years, numerous applications of metabolomics to chemical safety assessment have been proposed (e.g., mode-of-action (MoA) discovery as well as chemical grouping and read-across), although regulatory acceptance of metabolomics studies has yet to be achieved (van Ravenzwaay et al. 2012, 2016; Van den Eede et al. 2015; Cuykx et al. 2018; Dubuis et al. 2018; Ramirez et al. 2018; Zampieri et al. 2018). Indeed, this topic has previously been addressed by numerous review papers, workshops, and working groups (Buesen et al. 2017; Sauer et al. 2017; Viant et al. 2019; Harrill et al. 2021; Olesti et al. 2021).

In classical toxicology, point-of-departure (POD) derivation is employed alongside assessment factors for in vivo hazard characterisation to determine reference values, i.e., the maximum dose or concentration of a chemical within a defined exposure time frame without a significant risk to an individual (More et al. 2021). Calculating POD values from in vitro experiments is an attractive option to increase throughput of toxicological experiments at lower cost, and without the need for vertebrate testing. However, this approach additionally requires extrapolation of in vitro concentrations to corresponding in vivo doses (Sand et al. 2017). Whilst there have been many reports of successfully applying transcriptomics for POD derivation, similar studies applying metabolomics are scarce. van Ravenzwaay et al. compared no observed adverse effect level (NOAEL) values between data obtained using classical toxicology and metabolomics in rats exposed to a range of chemicals (van Ravenzwaay et al. 2014). The authors concluded that sensitivity of metabolomics (i.e., a lack of a previously established change in metabolite levels associated with the adverse effect) was comparable to classical toxicology in 75% of the investigated cases, providing further evidence for the potential of metabolomics in deriving PODs.

Benchmark concentration (BMC) modelling offers several advantages over the traditional NOAEL approach: (1) it is not limited to chemical concentrations used in the laboratory study, and (2) does not depend on the sample size, whilst (3) accounting for the variability of the data. Nonetheless, the BMC approach is computationally intensive and requires careful consideration of a benchmark response (BMR), i.e., the concentration or dose that results in a predefined change (e.g., 10% change in the response) thus producing the POD (benchmark concentration in vitro, or dose in vivo (BMC or BMD, respectively)) (US EPA 2012; Haber et al. 2018; Crizer et al. 2021). Most recently, BMC modelling was applied by Crizer et al. (2021) for metabolic footprinting of the HepaRG cell line exposed to liver and non-liver toxicants, in addition to transcriptomics analysis. In this case, the authors applied a BMR of one standard deviation, relative to the vehicle control samples. The study highlighted the value of applying untargeted metabolomics for this purpose, although the challenge of metabolite identification hindered data interpretation.

To date, there is no literature addressing the effect of sampling time on derivation of PODs when using untargeted metabolomics data. The overarching aim of this study was to evaluate how the sampling time affects the BMC values obtained from a high-throughput in vitro metabolomics study using nanoelectrospray direct infusion mass spectrometry (nESI-DIMS). To address this aim, it was first necessary to build the HepaRG-specific library of polar metabolites and lipids to maximise the confidence of annotating the nESI-DIMS data. The library was prepared by analysing cell extracts by ultra-high-performance liquid chromatography–mass spectrometry (UHPLC–MS/MS) using an Orbitrap ID-X Tribrid mass spectrometer with an AcquireX intelligent data acquisition workflow (Thermo Fisher Scientific). Next, HepaRG cells (5 × 10^4^ hepatocytes per well of a 96-well microplate) were individually exposed to four toxicants (aflatoxin B_1_, benzo[a]pyrene, cyclosporin A, or rotenone) across seven concentrations of each chemical and five sampling time points. To investigate the effects of sampling time, three methods were applied to determine the BMC values of the most sensitive features associated with the chemical exposure: (1) the 1st rank-ordered unannotated feature (i.e., the feature with the lowest BMC value), (2) the 1st rank-ordered putatively annotated feature with the use of a HepaRG-specific library of polar metabolites and lipids, and (3) the 25th rank-ordered feature, a method previously proposed for derivation of PODs using transcriptomics datasets (Reardon et al. 2021). Each method was applied to a dataset corresponding to a 24-h exposure period to allow sufficient time for development of a perturbation to the HepaRG cell line. Upon identifying a reliable method, the effect of sampling time on POD derivation was investigated.

## Materials and methods

### Cell culture and exposure

Undifferentiated HepaRG cells (HPR101, Biopredic International, Rennes, France, batches HPR-101056 and HPR-10101067) were cultured and exposed to chemicals according to the protocol described previously (Joossens et al. 2019). The preparation of the HepaRG-specific library of metabolites and lipids involved culturing hepatocytes of HepaRG incubated in 0.1% dimethyl sulfoxide (DMSO) for 24 h. For the toxicometabolomics study, hepatocytes of HepaRG were treated with one of four chemicals: aflatoxin B_1_ (purity ≥ 98.0%, Sigma), benzo[a]pyrene (purity ≥ 96.0%, Sigma), cyclosporine A (purity > 97.0%, Tokyo Chemical Industry) or rotenone (purity > 95.0%, Tokyo Chemical Industry), and subsequently sampled for metabolomics analyses across five time points following the onset of exposure (2, 6, 12, 24, and 48 h). Chemical stocks were prepared in DMSO (Sigma) and diluted in cell media using Hamilton Star and Starlet robotic platforms (Hamilton Italia Srl, Agrate, Brianza, Italy). The concentration range of aflatoxin B_1_, benzo[a]pyrene, and cyclosporine A was from 50 to 0.2048 μM (following 2.5 × dilution), whilst the concentration range of rotenone was from 10 to 0.04096 μM (also following 2.5 × dilution). After the exposure period has passed, cell media were removed and the cells were washed with ice-cold sodium chloride [2 × 180 μL (w/v; Fresenius Kabi, Isola della Scala, Italy)] and water [1 × 200 μL (sterile-filtered, BioReagent, Sigma or LC–MS grade, LiChrosolv, Sigma)] using ELx405 microplate washers (BioTek Instruments, Winooski, VT, USA), as previously proposed by Deng et al. (2018). This was followed by sealing the microplates using an X-Seal Manual Variable Temperature Thermal Sealer (Biorad foil, 180 °C, 5 s) and placing them on dry ice. Once frozen, the microplates were stored at − 80 °C until shipment from Italy to the United Kingdom.

The toxicometabolomics study included three technical (i.e., samples on the same 96-well microplate corresponding to the same biological replicate) and three biological (i.e., samples on different 96-well microplates which were split during the culturing process) replicates for treated cells per time point, comprising nine samples in total per chemical and time point. There were also nine technical and three biological replicates for negative control samples per time point (twenty-seven samples in total per time point), corresponding to cells incubated in media with 0.1% DMSO. In addition, three wells on each microplate corresponded to hepatocytes exposed to rotenone at 4 μM. Intrastudy quality control (QC) samples were comprised of hepatocytes cultured and treated the same way as the study samples corresponding to 2 and 48 h time points and three biological replicates. The extraction blanks were prepared in the same manner but did not contain biological material. A subset of the data from this study, corresponding to negative control samples at 24 h, was analysed and published previously alongside the methods applied (Malinowska et al. 2022a).

### Metabolite extraction

Metabolite extraction was conducted with the use of Biomek i7 Hybrid Workstation (Beckman Coulter) as described previously (Malinowska et al. 2022a, b). The deck included automated labware positions pre-cooled to − 15 °C for metabolite extraction or 4 °C for sample resuspension prior mass spectrometry analysis. Polar metabolites were extracted with the use of pre-cooled 4:1 (v/v) methanol:water containing 1.5 μM tryptophan-d_5_ (isotopic purity of 97 atom % deuterated, Sigma) with samples in two 96-well microplates prepared in parallel. Methanol (LC–MS grade) and water (LC–MS grade) were purchased from Honeywell (Charlotte, NC, USA) and Merck (Darmstadt, Germany), respectively. In brief, 60 μL of the extraction solvent was added to each well of a 96-well microplate containing washed and frozen cells, followed by mixing well contents and transfer of 40 μL aliquots per well to a new 96-well microplate (a collection microplate, Eppendorf, Hamburg, Germany). Next, 60 μL per well was added to the first 96-well microplate and the same volume was transferred to the collection microplate. The steps were then repeated for the extraction of metabolites from the second 96-well microplate with washed and frozen cells. Next, the samples were shaken (200 rpm, 2 min, room temperature) and centrifuged (3622*g*, 3 min, 4 °C, Sigma 6-16KL) with 80 μL of supernatant per well transferred for drying with the use of a SPD111V230 concentrator (Thermo Scientific Savant) for 2 h at 35 °C. Dried samples were sealed with an ALPS 50 V-manual heat sealer from Thermo Scientific (165 °C, 1.5 s) and stored at − 80 °C until mass spectrometry analysis.

Lipids were extracted by adding 60 μL of pre-cooled methanol containing 1.25 μM dodecylphosphorylcholine-d_38_ (isotopic purity of 98 atom % deuterated, Sigma) per well followed by mixing well contents and transfer of 40 μL aliquots per well to a collection microplate (Eppendorf, Hamburg, Germany). Forty μL aliquots of methanol containing the deuterated internal standard were then added again to the original microplate and the same volume was transferred to the collection microplate followed by an addition of 40 μL of chloroform (HPLC-grade, ≥ 99.8% stabilised with 2-methyl-2-butene, VWR Chemicals). The steps were then repeated for lipid extraction from the second 96-well microplate with washed and frozen cells. Shaking and centrifugation were conducted as described above for polar metabolites. Following centrifugation, 96 μL of supernatant per well was transferred for drying with the use of nitrogen blowdown (Techne Dri-Block DB100/3 sample concentrator) for approximately 15–20 min at 35 °C. Subsequent sealing of dried samples and their storage was conducted as described for polar metabolites.

For the preparation of the HepaRG-specific library of polar metabolites and lipids, metabolite extracts from individual wells of 96-well microplates were pooled into a polypropylene reservoir (Beckman Coulter, maximum capacity 38 mL). The pool was mixed and aliquots of 1600 μL (polar metabolites) or 1632 μL (lipids) were transferred to Eppendorf tubes for polar metabolites or 1.75 mL glass vials for lipids (glass screw neck specimen vials, Fisherbrand) followed by drying as described above (with the exception of drying time due to a larger sample volume).

### Metabolomics data acquisition

#### HepaRG-specific library of polar metabolites and lipids

Polar metabolite and lipid extracts were thawed on wet ice for 15 min (*n* = 3 for each UHPLC–MS/MS assay and ionisation mode) and resuspended in ice-cold 100 μL of 1.5:1.5:1 (v/v/v) acetonitrile:methanol:water or 100 μL of 3:1 (v/v) propan-2-ol:water, respectively. The extracts were vortexed for 2 min and sonicated for 10 s, a process that was repeated three times in total. The resuspended samples were then centrifuged: 20,000*g*, 20 min, 4 °C (centrifuge model 5920 R, Eppendorf) for polar metabolites or 2500*g*, 10 min, 4 °C (model Primo R Centrifuge, Thermo Scientific) for lipids. The supernatants (80 μL) were transferred to UHPLC–MS/MS vials for a given assay and ionisation mode and maintained at 4 °C throughout the analytical run. The metabolome of the HepaRG cell line was analysed using UHPLC–MS/MS by applying hydrophilic interaction liquid chromatography (HILIC) and reversed-phase chromatography (RP) C_30_ methods published previously (D’Elia et al. 2019; Southam et al. 2020, 2021; Jankevics et al. 2021). Data collection was conducted using Vanquish UHPLC and Orbitrap ID-X Tribrid mass spectrometer with an AcquireX intelligent data acquisition workflow (Thermo Fisher Scientific). The Vanquish UHPLC was equipped with a column compartment (VH-C10-A), split sampler FT (VF-A10-A), and binary pump H (VH-P10-A). HILIC analyses employed an Accucore 150 Amide HILIC column (2.1 × 100 mm, particle size 2.6 μm, Thermo Fisher Scientific) with a column guard Accucore 150 Amide HILIC (2.1 × 10 mm, particle size 2.6 μm, Thermo Fisher Scientific) maintained at 35 °C in positive and negative ionisation modes. RP C_30_ analyses were conducted using an Accucore C_30_ column (2.1 × 150 mm, particle size 2.6 μm, Thermo Scientific) maintained at 55 °C in positive and negative ionisation modes. Mass spectrometry data was collected over an *m*/*z* range of 70–1050 (HILIC) or 150–2000 (RP C_30_). Detailed description of UHPLC–MS/MS methodology is provided in Supplementary Information (including Fig. S1 and S2).

#### Toxicometabolomics study

The method of data acquisition employing nESI-DIMS (termed “internal scan replication”) was a modification of the approach proposed by Southam et al. (2007, 2017) that was optimised for small biomass samples and further improved for high-throughput analyses (Malinowska et al. 2022a, b). The dataset for each nESI-DIMS assay was acquired using a TriVersa NanoMate coupled to an Orbitrap Elite mass spectrometer over five analytical batches with each sequence being composed of study samples, intrastudy QC samples, extraction blanks, and a synthetic mixture of metabolite standards of known composition.

Given the high-throughput capability of our metabolomics workflow, three study microplates and one microplate containing intrastudy QC samples and extraction blanks were resuspended for mass spectrometry analysis at a given time. Polar metabolites for nESI-DIMS analysis in positive ionisation mode were resuspended in 30 μL per well of pre-cooled 4:1 (v/v) methanol:water containing 0.25% (v/v) formic acid (~ 98%, Honeywell), whilst polar metabolites for nESI-DIMS analysis in negative ionisation were resuspended in 30 μL of pre-cooled 4:1 (v/v) methanol:25 mM aqueous ammonium acetate (≥ 99.9% trace metal basis, Honeywell). Lipids for nESI-DIMS analysis in positive ionisation mode were resuspended in 40 μL of pre-cooled 2:1 (v/v) 7.5 mM methanolic ammonium acetate:chloroform. The resuspended extracts were centrifuged (3622*g*, 3 min, 4 °C, Sigma 6-16KL) with the supernatant (20 μL per well) transferred to a 384-well microplate. The samples were centrifuged again in the 384-well microplate prior nESI-DIMS analysis (2000*g*, 10 min, 4 °C, Sigma 6-16KL). The three assays are referred to later in the manuscript as polar positive, polar negative and lipid positive nESI-DIMS assays, respectively.

### Data processing and analysis

#### HepaRG-specific library of polar metabolites and lipids

The data processing steps of samples were conducted using either Compound Discover (Thermo Scientific, version 3.2.0.421) for HILIC UHPLC–MS/MS analyses or LipidSearch (Thermo Scientific, version 4.2.29) for RP C_30_ UHPLC–MS/MS analyses. The preparation of the library involved spectral fragmentation matches (experimental MS^2^ and/or MS^3^) with the mzCloud library (polar metabolites) and LipidSearch (lipids). Detailed UHPLC–MS/MS methods for data processing and analysis are provided in Supplementary Information.

#### Toxicometabolomics study

Data processing consisted of peak picking (known as “process scans”) followed by correcting the drift in mass accuracy of the dataset. This drift was observed for the internal standard (putatively annotated) within each biological sample and confirmed by the analysis of a system suitability QC sample containing a mixture of metabolite standards (spanning a wide *m*/z range) that had been measured at the beginning and end of each analytical batch. The observed drift in mass accuracy was modelled and corrected by applying a 1-D smoothing spline and leave-one-out cross-validation for the putatively annotated feature of the internal standard (either [M + H]^+^ or [M − H]^−^). This step was followed by aligning features across all samples, and the removal of outlying samples identified by their intensity of the internal standard exceeding 3 × median absolute deviation away from the median. The removal of outliers was conducted separately for study samples and intrastudy QCs as described previously (Malinowska et al. 2022a). The data processing was rerun without the outliers followed by subtracting features present in extraction blanks and retaining only features present across 50% of the dataset for a given assay. Then, samples were removed which had a high percentage of missing values (≥ 40% for polar positive assay or ≥ 50% for polar negative and lipid positive assays) followed by the removal of features which were not present in at least 70% of intrastudy QCs. Next, signal drift and batch effect correction, probabilistic quotient normalisation, and removal of variable features with intensity RSDs > 30% in intrastudy QCs were performed. For principal component analysis (PCA), missing values were imputed using the k-nearest neighbour algorithm (*k* = 5) followed by a generalised log transformation and mean centring. The PCA was used to evaluate general trends in data and to identify any outliers. The outliers were removed using a 95% confidence interval, followed by re-processing the dataset without these outliers. Initial data evaluation of the whole dataset revealed that all the samples from a microplate corresponding to biological replicate one at 48 h were outliers (consistently for all the three assays). Thus, these samples were removed and the data reprocessed as aforementioned. All data processing were conducted using DIMSpy tools within the Galaxy workflow management system and R/Bioconductor structToolbox (version 1.4.2) (Southam et al. 2017; Lloyd et al. 2020; Weber and Zhou 2020).

For benchmark concentration analysis, the dataset was sub-grouped, retaining only samples corresponding to a single chemical exposure (in addition to unexposed control samples), time point and nESI-DIMS assay. Next, only features present in at least four or five samples in each concentration group were retained. This value (i.e., *n* = 4 or *n* = 5) was dependent on whether the maximum percentage of missing values exceeded 50%, i.e., if the filter specifying that a feature must be present in at least four samples in each concentration group resulted in the percentage of missing values above 50%, the value for that filter was adjusted from 4 to 5. This procedure was necessary as the modelling required imputation of missing values using k-nearest neighbour algorithm, allowing a maximum of 50% of missing values per feature or sample. These steps were conducted using R/Bioconductor structToolbox (version 1.6.0) (Lloyd et al. 2020). Table S1 in Supplementary Information contains the maximum percentage of missing values per feature, sample and feature count for datasets with samples exposed to a given chemical (and respective negative control samples) at a selected time point and measured by one of three nESI-DIMS assays. Benchmark concentration modelling was applied to scaled data using BMDExpress [version 2.30.050 (Phillips et al. 2019)] implemented via Birmingham Environment for Academic Research (BlueBEAR, Linux High Performance Computing environment), and CaStLeS (Compute and storage for the Life Sciences) (Thompson et al. 2019). Benchmark concentration modelling was conducted twice: first, the BMR was set to three standard deviations (SD) which served as a filter to retain only features demonstrating some degree of a concentration–response relationship. The features were fit to the following models: Exponential (2nd to 5th order), Linear, Hill and Power. The confidence level was 0.95, constant variance was on, power restricted (≥ 1), and maximum iterations were set to 250. Model selection had the following criteria: benchmark model concentration at lower confidence limit (BMCL) and upper bound (BMCU) were computed ignoring non-convergence. Best poly model test was based on the lowest Akaike information criterion, whilst p-value threshold was set to 0.05. Hill model with ‘*k*’ parameter below 1/3 of lowest positive dose was flagged and excluded from best models. This process was repeated with identical settings aside from a BMR value that was changed from 3 to 1 SD. Only features passing selected criteria for both analyses at 3 and 1 SD were retained. These criteria were as follows: BMC values had to be smaller than the highest concentration of a chemical in the given dataset, best model *p* value had to be greater than 0.0001 and BMC/BMCL value had to be less than 20.

### Metabolite annotation

Metabolite annotation was conducted using the Python package BEAMSpy (Birmingham mEtabolite Annotation for Mass Spectrometry, https://github.com/computational-metabolomics/beamspy, version 1.1.0). The database employed for putative metabolite annotation was an in-house HepaRG-specific library of metabolites and lipids prepared using spectral fragmentation matches (experimental MS^2^ and/or MS^3^) with the mzCloud library (polar metabolites) or LipidSearch (lipids), where the experimental data were derived from UHPLC–MS/MS analyses of the HepaRG cell line using a Vanquish UHPLC and Orbitrap ID-X Tribrid mass spectrometer with an AcquireX intelligent data acquisition workflow (Thermo Fisher Scientific). The list of metabolites and lipids used for putative annotations of datasets is included in the Supplementary Table S2 A–C. The mass error employed for putative annotation was ± 5 ppm, and the adduct list included [M + H]^+^, [M + Na]^+^, [M + NH4]^+^ for positive ionisation mode, and [M − H]^−^, [M + Cl]^−^, [M + Hac − H]^−^ for negative ionisation mode. The putatively annotated spectral features with concentration–response behaviour identified by benchmark concentration modelling are included in Supplementary Tables S3–S6.

## Results and discussion

### HepaRG-specific library of polar metabolites and lipids

To maximise the confidence of annotating HepaRG toxicometabolomics datasets, it was first necessary to characterise the detectable metabolome and lipidome of this cell line. The curated and filtered list, forming the HepaRG-specific library of polar metabolites and lipids, is applicable to the current and future metabolomics studies, in particular for DIMS experiments where metabolite annotation is particularly challenging given the lack of chromatographic separation.

Following spectral matching, 54 polar metabolites were annotated in positive ionisation mode with MS^2^ mass spectra, of which 33 polar metabolites also yielded MS^3^ fragments (the quality of MS^3^ fragments was evaluated manually) (Table 1). It should be noted that version 3.2 of Compound Discoverer only provides spectral matches using MS^2^ (not MS^3^) data. Fourteen polar metabolites were annotated in negative ionisation mode with MS^2^ mass spectra, with 2 of these metabolites producing MS^3^ fragments. In total, 10 metabolites were annotated in both positive and negative ionisation. The measurements described here meet the criteria for Metabolomics Standards Initiative (MSI) level 2 annotations, which entails a match using physicochemical properties and/or a match to a spectral library (Sumner et al. 2007, 2014). It should be noted, however, that for some *m/z* features more than one spectral match was reported by Compound Discoverer and the mzCloud library, which limits the confidence of that annotation. For example, the same *m/z* features were putatively annotated as l-leucine, l-isoleucine and l-norleucine, making assignment of the level of metabolite annotation proposed by Sumner et al. (2007, 2014) somewhat challenging.

**Table 1:** Summary of polar metabolites and lipids measured in the extracts of the HepaRG cell line using HILIC and RP C_30_ UHPLC–MS/MS assays in positive and negative ionisation modes

Dataset (assay and ionisation mode)	Number of metabolites		
	Based on MS^2^ fragmentation data	Based on MS^2^ and MS^3^ fragmentation data	Present in MTox700+
HILIC positive	54	33	37–41
HILIC negative	14	2	11

Dataset (assay and ionisation mode)	Number of lipids		
	Grade ‘BB-’ or higher	With unique molecular formulae	Present in MTox700 +
RP C_30_ positive	246	194	6–24
RP C_30_ negative	107	84	2–7

Range refers to the worst- and best-case scenarios of a metabolite being present in the MTox700+ biomarker list (Sostare et al. 2022), where a metabolite is excluded or included from the count if it has multiple annotations, respectively

Metabolites present in the HepaRG-specific library were searched against the MTox700+ biomarker list (Sostare et al. 2022), which contains toxicologically relevant polar metabolic and lipid biomarkers obtained through interrogation of existing literature, databases, and analytical assays. These biomarkers should in principle be equivalent to panels of genes used in transcriptomics studies such as the S1500+ panel developed by the US National Toxicology Program (Mav et al. 2018). In Table 1, worst- and best-case scenarios are shown, where a metabolite is excluded or included from the count if it has multiple annotations, respectively, e.g., a putative annotation of adenosine 3’-monophosphate (absent from the MTox700+ panel) represents the ‘worst case’ scenario, whilst adenosine 5’-monophosphate (present in the MTox700+ panel) represents the ‘best case’ scenario. The majority of metabolites detected in polar extracts of the HepaRG cell line are present in the MTox700+ list, highlighting the toxicological-relevance of the HILIC UHPLC–MS/MS data. The polar metabolites measured in HepaRG were assigned a subclass based on the information retrieved from the Human Metabolome Database (HMDB), version 4.0 (Wishart et al. 2018) as shown in Fig. S3. For metabolites measured in positive ionisation mode, amino acids, peptides and analogues were the most abundant (22 out of 54 metabolites were assigned this subclass), as anticipated when using HILIC UHPLC–MS/MS, which separates small hydrophilic metabolites (Greco and Letzel 2013). Reduced and oxidised glutathione are examples of biomarkers present in this subclass: a ratio of reduced to oxidised glutathione is an important biomarker of oxidative stress (Vairetti et al. 2021). Carnitine (belonging to a subclass of quaternary ammonium salts) also has important biological functions, such as participation in β-oxidation of long-chain fatty acids (Li and Zhao 2021).

To maximise the confidence of lipid annotations in the HepaRG cell line, only lipids of grade ‘BB- ‘ (or higher) across 3 technical replicates were retained, as shown in Table 1 (comparable to a lipid being present in at least 2 out of 3 replicates). The grades assigned by LipidSearch reflect the quality of the annotation, i.e., grade B denotes a lipid molecule with an assigned class and some fatty acid chains (Kiyonami et al. 2016). There were 246 and 107 lipid molecules found in positive and negative ionisation modes, respectively, with an annotation quality grade of ‘BB-’ or higher (Table 1). It should be noted that lipid molecules were not assigned the following information: (1) stereochemical number (*sn*-position for derivatives of glycerol), (2) double-bound location, and (3) stereochemistry. If the *sn*-position is not assigned, the name of the lipid molecule contains an underscore, e.g., PC 16:0_18:1 (Valenzuela and Valenzuela 2013; Peake et al. 2019). This makes the assignment of MSI levels to the lipids challenging. Next, the number of lipids with unique molecular formulae was determined, yielding 194 lipids in positive ionisation mode, and 84 lipids in negative ionisation mode. In UHPLC–MS/MS experiments, lipid molecules that share the same molecular formula may be chromatographically separated and fragmented; however, this is not the case when conducting high-throughput DIMS experiments since these compounds cannot be differentiated using only accurate mass from MS^1^ experiments.

The number of lipids present in the MTox700+ biomarker list was determined using worst- and best-case scenarios (Table 1). As anticipated, there were few lipid molecules detected that overlapped with MTox700+ , mostly because this biomarker list contains relatively few lipids. These low values are not surprising considering a) the diversity of lipidome and b) that not every putative annotation of a lipid molecule has a corresponding HMDB ID, which is the unique identifier used when searching the MTox700+ metabolite panel (Hu et al. 2019; Sostare et al. 2022). Next, the putatively annotated lipids were assigned lipid classes according to LipidSearch, as shown in Fig. S4. Phosphatidylethanolamines, triglycerides and phosphatidylcholines were the top three most abundant lipid classes found in positive ionisation mode. These findings are in agreement with existing literature, as phosphatidylcholines and phosphatidylethanolamines (commonly found in cell membranes) are the most abundant glycerophospholipids, and have been associated with non-alcoholic fatty liver disease (NAFLD), Alzheimer’s and Parkinson’s diseases (Fagone and Jackowski 2013; Calzada et al. 2016). Triglycerides, which are stored in the liver, have also been linked to NAFLD (Alves-Bezerra and Cohen 2018). In negative ionisation mode, phosphatidylethanolamines, phosphatidylinositols, and phosphatidylserines were the top three most abundant classes of lipids detected, followed by phosphatidylglycerols and ceramides.

The use of the curated HepaRG-specific library in experiments involving DIMS data is anticipated to be a valuable tool, providing researchers with greater confidence in the annotation of spectral features when using only MS^1^ accurate mass measurements. Whilst a useful tool, it should be noted that the knowledge of the human metabolome is continuously developing as is its toxicological relevance, and therefore such curated lists cannot provide exhaustive annotations and undoubtedly require periodic updates.

### Toxicometabolomics study—assessment of methods for POD derivation

Prior to assessing methods for POD derivation, the data quality was evaluated by determining the feature count, variability of intrastudy QC sample (repeatedly measured throughout each assay), and variability of negative control samples at each sampling time point (Table S1). All three parameters assessed were highly satisfactory with median relative standard deviation (mRSD) below the threshold of 30% previously suggested by the community (Viant et al. 2019). To address the effect of sampling time points (i.e., exposure time) on BMC values obtained from high-throughput in vitro metabolomics data, it was necessary to initially investigate approaches for selecting the most robust metabolic features for POD derivation. The transcriptomics community has explored this topic in depth by evaluating several approaches to select groups of genes and/or molecular pathways to derive PODs (Farmahin et al. 2017; Ramaiahgari et al. 2019; Reardon et al. 2021); however, some of these methods (e.g., pathway analysis) require robust annotation and identification of the ‘features’ being measured (i.e., metabolites in this study), which remains a challenge for untargeted metabolomics workflows (Nash and Dunn 2019). Given that the current dataset was obtained using nESI-DIMS (i.e., lacking chromatographic separation), only putative annotations of metabolites and lipids based upon accurate *m*/*z* of full scan data (MS^1^ data) were possible. Consequently, the three POD derivation methods applied in this study were focused on individual mass spectral features, metabolites and/or lipids, not on pathway-based approaches, with the focus on the most sensitive mass spectral features responding to chemical exposure. These approaches took into consideration both unannotated spectral features (with the lowest BMC values, but possibly lacking metabolite annotation) as well as putatively annotated spectral features (often providing higher BMC values in comparison to the former approach, but in principle placing the findings in a biological context), as discussed below.

**Fig. 1:**
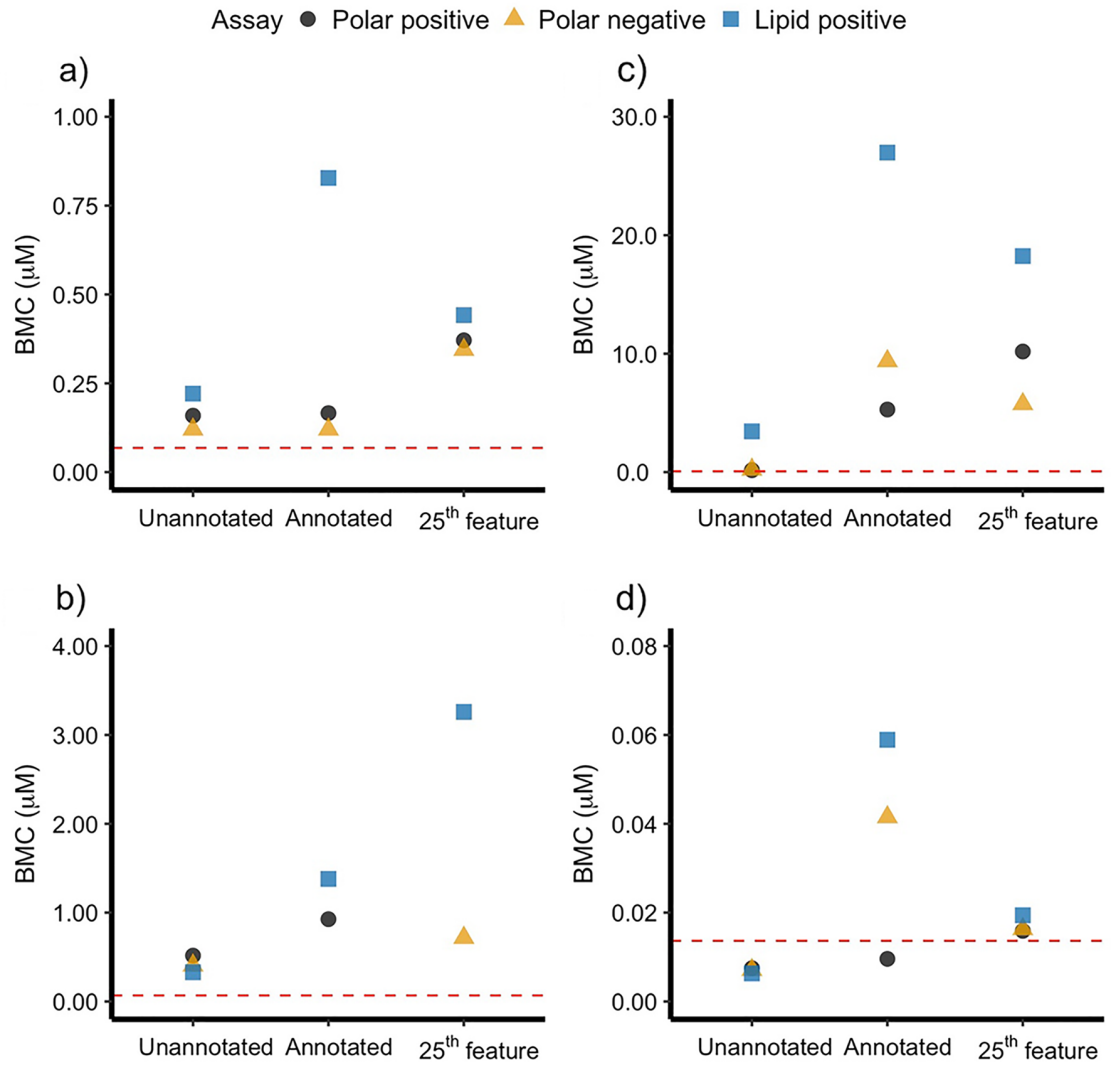
Assessment of methods for POD derivation from BMC modelling using high-throughput in vitro metabolomics data. The HepaRG cell line was exposed to **a** aflatoxin B_1_, **b** benzo[a]pyrene, **c** cyclosporin A, and **d** rotenone for 24 h. The three approaches evaluated were (1) the 1st rank-ordered unannotated feature (i.e., the feature with the lowest BMC value) termed “Unannotated”, (2) the 1st rank-ordered putatively annotated feature (with putative annotations derived using the HepaRG-specific library of polar metabolites and lipids) termed “Annotated”, and (3) the 25th rank-ordered feature termed “25th feature”. The colours and shapes used for plotting the data indicate nESI-DIMS assays used for measurement of metabolites and lipids. The red dashed line indicates the lower limit of extrapolation as suggested by the National Toxicology Program (i.e., 1/3 of the lowest experimental concentration in the study) (Auerbach et al. 2018)

The three methods assessed here were (1) the 1st rank-ordered unannotated feature (i.e., the feature with the lowest BMC value), (2) the 1st rank-ordered putatively annotated feature (with putative annotation conducted using the HepaRG-specific library of polar metabolites and lipids), and (3) the 25th rank-ordered feature previously proposed for derivation of PODs using transcriptomics datasets (Reardon et al. 2021). These approaches were evaluated for the four chemicals to which the HepaRG cell line was exposed over 24 h, and are referred to in Fig. 1 as “Unannotated”, “Annotated”, and “25th feature”, respectively. As expected, the lowest BMC values were obtained using the 1st rank-ordered unannotated feature. For aflatoxin B_1_, the most sensitive feature measured by polar nESI-DIMS assay in negative ionisation mode was also putatively annotated, however, that was the only chemical-assay combination for which the most sensitive feature could be named. Whilst the differences between BMC values obtained for each method were less than tenfold for the datasets corresponding to aflatoxin B_1_ (Fig. 1a) and benzo[a]pyrene (Fig. 1b), the dataset with samples exposed to cyclosporin A revealed more striking differences between these methods (Fig. 1c). For polar metabolites measured in positive and negative ionisation modes, the differences between the 1st rank-ordered unannotated feature, the 1st rank-ordered putatively annotated feature, and 25th rank-ordered feature exceeded tenfold when comparing the BMC values. This larger difference may stem from the relatively small size of the HepaRG-specific library for the “annotated” approach, which was employed in this study to decrease the rate of false positive annotations. The 25th rank-ordered feature also yielded higher BMC values than those derived using the 1st rank-ordered unannotated feature. In principle, this method offers more stable POD derivation; however, the choice of the 25th rank-ordered feature is somewhat arbitrary and unexplored for metabolomics. Lastly, the analysis of rotenone dataset revealed very low BMC values across all methods, all of which were within tenfold of each other. Thus, whilst for the majority of chemicals (aflatoxin B_1_, benzo[a]pyrene, rotenone) the three POD derivation approaches generated BMC values differing by less than tenfold (per chemical), PODs corresponding to the dataset with cyclosporine A revealed much larger differences. Consequently, to study the effect of sampling time, the approach using the 1st rank-ordered unannotated feature was employed, which was not biased by the effectiveness of annotating features using the HepaRG-specific metabolite library. The findings presented here are mostly in agreement with findings presented by Farmahin et al. (2017) who concluded that several approaches examined in that study were appropriate for POD derivation using transcriptomics, especially if working with datasets across multiple sampling time points. In addition, the BMD values from transcriptomics datasets were well aligned with apical PODs (less than tenfold of each other) further highlighting the value of omics studies in this area. On the other hand, Crizer et al. (2021) proposed the use of a metric termed “the lowest consistent response dose” (LCRD) to determine the most sensitive features that are likely to be toxicologically relevant. This approach also used unannotated mass spectral features with concentration–response behaviour rather than relying on putative metabolite annotations.

### Toxicometabolomics study—investigation of sampling time points on POD values

**Fig. 2:**
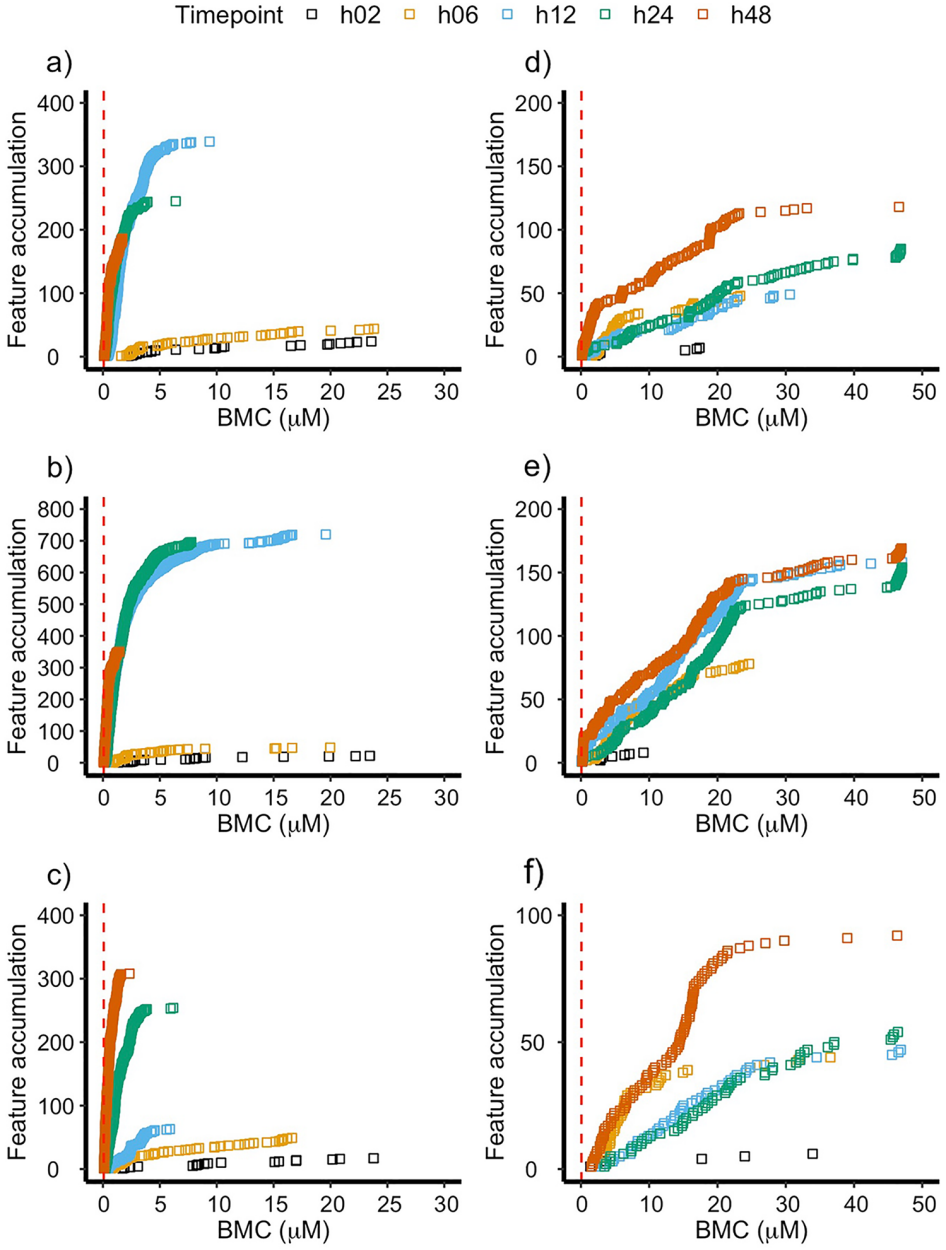
Accumulation plots corresponding to the exposure of the HepaRG cell line to aflatoxin B_1_ (**a**–**c**) and cyclosporin A (**d**–**f**) at five sampling time points. The metabolome was measured by polar positive (**a**, **d**), polar negative (**b**, **e**) and lipid positive (**c**, **f**) nESI-DIMS assays. The plots demonstrate accumulation of features with concentration–response behaviour ordered by the lowest BMC value. The red dashed line indicates the lower limit of extrapolation as suggested by the National Toxicology Program (i.e., 1/3 of the lowest experimental concentration in the study) (Auerbach et al. 2018)

**Fig. 3:**
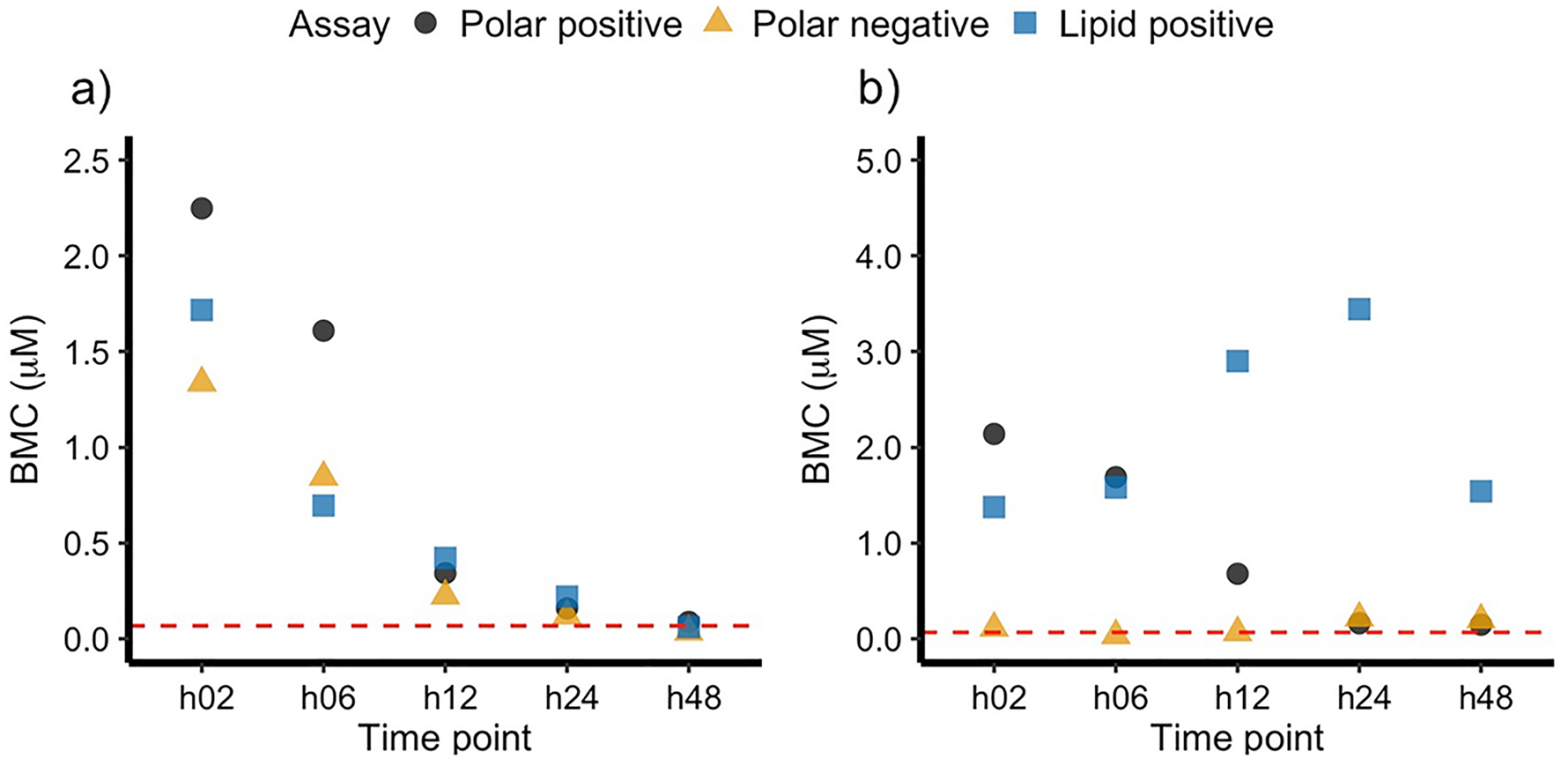
Plots demonstrating the effect of exposure time on BMC values derived from the metabolic responses of the HepaRG cell line exposed to **a** aflatoxin B_1_, and **b** cyclosporin A, obtained by three nESI-DIMS assays measuring the polar metabolome and lipidome. The approach used to derive the BMC value for each time point and assay corresponds to the 1st rank-ordered unannotated feature. The red dashed line indicates the lower limit of extrapolation as suggested by the National Toxicology Program (i.e., 1/3 of the lowest experimental concentration in the study) (Auerbach et al. 2018)

The effect of time on the metabolome of HepaRG perturbed by aflatoxin B_1_ was studied at 2, 6, 12, 24 and 48 h. The use of accumulation plots allowed for visualisation of metabolic features with concentration–response behaviour ordered by increasing BMC values measured by polar positive (Fig. 2a), polar negative (Fig. 2b), and lipid positive (Fig. 2c) nESI-DIMS assays. The number of features with concentration–response behaviour increased over early time points from tens to hundreds (2, 6 and 12 h) followed by their decrease at 24 and 48 h for the case of the polar metabolome (in both ionisation modes), whilst this number of features increased over five sampling time points for features detected by the lipid positive nESI-DIMS assay. The BMC values consistently decreased with longer exposure times across the three assays confirming the anticipated decrease in POD with increasing exposure time. This observation was confirmed when plotting the 1st rank-ordered unannotated feature for each sampling time point and assay (Fig. 3a). A time-dependent effect on BMC values was evident, which was consistently observed across the DIMS assays employed, with the values decreasing 25-, 38-, and 28-fold for polar positive, polar negative, and lipid positive assays respectively, when comparing the earliest (2 h) and latest (48 h) sampling time points. To understand how exposure to aflatoxin B_1_ affects the metabolome of HepaRG, putative annotations of the spectral features were used. The analysis of features with concentration–response behaviour revealed that several triglycerides and ceramides increase their spectral intensities in response to increasing concentrations of aflatoxin B_1._ This was observed for triglycerides and ceramides measured across multiple sampling time points and assays in the study, and it is a known marker of steatosis (Cuykx et al. 2018).

**Fig. 4:**
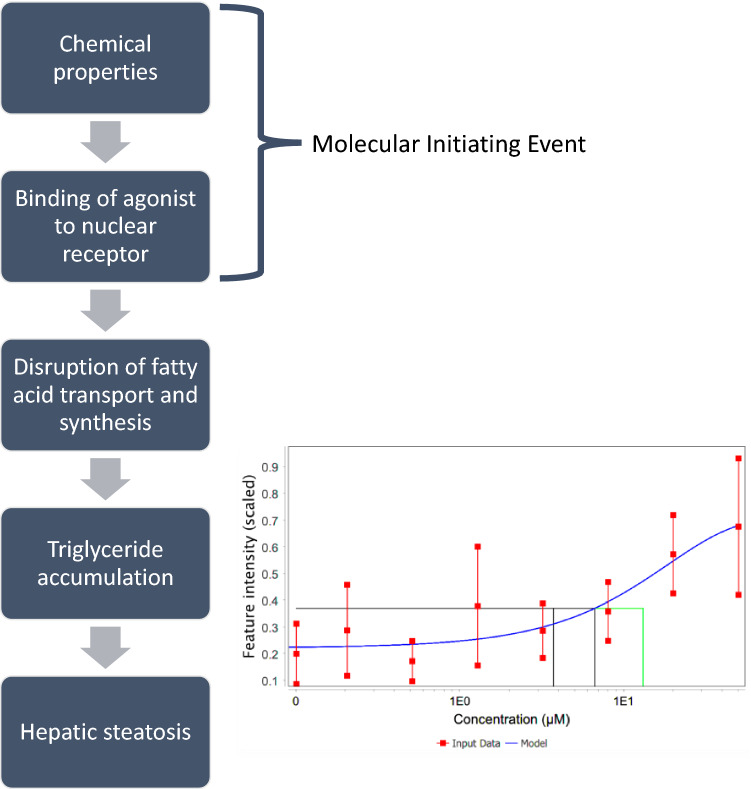
The adverse outcome pathway for steatosis reproduced from Mellor et al. and Vinken et al. (Mellor et al. 2016; Fig. 1; Vinken 2016; Fig. 2). The molecular key event for steatosis is the accumulation of triglycerides (TG), which was measured by untargeted metabolomics (lipid positive nESI-DIMS assay) in the HepaRG cell line exposed to cyclosporin A for 48 h. The putative annotation of this feature corresponds to TG(18:0_18:1_20:3)/TG(20:1_18:1_18:2). The BMC value for this TG was 6.6 μM

Exposure of the HepaRG cell line to cyclosporin A revealed that the latest time point (48 h) was associated with the highest number of features with concentration–response behaviour (Fig. 2d–f), however the effect of sampling time points on BMC values was less apparent in comparison to the dataset with aflatoxin B_1_. This could be further observed in Fig. 3b, where the 1st rank-ordered unannotated feature was plotted for every time point and assay. Whilst the BMC values decreased over time for features measured by the polar positive nESI-DIMS assay, the opposite was true for the lipid positive assay for time points 2, 6, 12 and 24 h, whilst the BMC values for features measured by the polar negative assay fluctuated over 48 h. It is noteworthy that measurement of the polar metabolome at 48 h using positive and negative ionisation modes demonstrated consistency in BMC values, which were within only 1.3-fold of each other (0.15 and 0.20 μM, respectively), whilst the BMC value for a lipid feature was higher (1.54 μM). Given existing literature describing the toxicity of cyclosporin A, which demonstrated that exposure to this substance could result in steatosis and cholestasis, it was possible to place the newly reported BMC values in a biological context by incorporating them within an adverse outcome pathway (AOP) for steatosis (Vinken et al. 2013; Joossens et al. 2015; Mellor et al. 2016; Vinken 2016; Angrish et al. 2017). One of the molecular key events for steatosis is accumulation of triglycerides (TG), a class of lipids that was measured in this study by lipid positive nESI-DIMS (Fig. 4). One mass spectral feature exhibiting concentration–response behaviour was a triglyceride (*m*/*z* 928.83266) putatively annotated as TG(18:0_18:1_20:3)/TG(20:1_18:1_18:2) with a mass error of 0.1 ppm and BMC value of 6.6 μM; this feature demonstrated its increase with an increasing concentration of cyclosporin A (Fig. 4). Whilst the BMC value of this triglyceride was 4.3-fold higher than that obtained using the 1st rank-ordered unannotated feature for the lipid positive assay at 48 h, its annotation enables a more mechanistically anchored interpretation of this POD based on an established AOP. Thus, this example highlights the importance of continued development of the AOPs, also for subsequent use in POD derivation.

The dataset corresponding to benzo[a]pyrene, a procarcinogen (Fig. S5a–c), demonstrated fewer features with concentration–response behaviours at earlier time points (2, 6 and 12 h) indicating the anticipated increased potency of this chemical with exposure time. Consequently, the comparison of BMC values at five time points was less informative, as the polar positive nESI-DIMS assay did not reveal any features with concentration–response behaviour at 2, 6 and 12 h, which was also observed for the lipid positive assay at 2 h (Fig. S6a). For polar negative and lipid-positive nESI-DIMS assays, the BMC value for the 1st rank-ordered unannotated feature increased over 12 h, followed by its subsequent decrease. Later time points (24 and 48 h) demonstrated consistency in the BMC values obtained by three separate nESI-DIMS assays, being within 1.6-fold of each other. The putatively annotated feature with the second lowest BMC value of 0.93 μM corresponded to l-kynurenine ([M + H]^+^, mass error: 0.5 ppm) measured by the polar positive assay at 24 h. For this spectral feature, it was observed that with an increasing concentration of benzo[a]pyrene, its intensity decreased at the 24-h sampling time point. l-kynurenine is a member of the kynurenine pathway, an important metabolic pathway in cancer studies, where l-tryptophan is catabolised. In addition, l-kynurenine is an agonist of aryl hydrocarbon receptors, which benzo[a]pyrene binds to (Shiizaki et al. 2017; Krishnamurthy et al. 2021).

Lastly, rotenone (an inhibitor of mitochondrial complex I, and a model toxicant in studies of oxidative stress) was shown to be highly potent leading to low BMC values (mostly below 1 μM), an effect that was consistently observed for all time points and assays (Fig. S5d-f) (Mennecozzi et al. 2012; Gielisch and Meierhofer 2015). The effect of sampling time point was apparent in terms of the number of features with concentration–response behaviour for polar negative and lipid positive nESI-DIMS assays with the value following the increasing exposure time. Fig. S6b employing only the 1st rank-ordered unannotated feature for each assay and time point demonstrated an overall trend with the BMC values for these features increasing slightly from 2 to 24 h, followed by their decrease at 48 h. All of these values were below the lower limit of extrapolation suggested by the National Toxicology Program (i.e., 1/3 of the lowest experimental concentration in the study) (Auerbach et al. 2018).

## Conclusions

Untargeted metabolomics is being recognised as a potential tool for use in supporting chemical safety assessment, including POD derivation through BMC modelling. This study demonstrated the value of high-throughput in vitro metabolomics, allowing investigation of three approaches to derive BMC values from chemical-exposed HepaRG cell line, and subsequently to apply BMC modelling to study the effect of exposure time on POD derivation. In particular, it was shown that for three out of the four chemicals studied, all approaches employed (the 1st rank-ordered unannotated feature, the 1st rank-ordered putatively annotated feature, and the 25th rank-ordered feature) led to relatively consistent BMC values that differed by less than tenfold (per chemical). Whilst the approach of using the 1st rank-ordered unannotated feature offers the most sensitive response to chemical exposure, the lack of annotation impedes the interpretation of the biological findings (e.g., attempting to link it to a MoA). Similarly, the 25th rank-ordered feature might suffer from a similar problem; however, it at least offers a more stable POD. It should be noted that the choice of the 25th rank-ordered feature is subjective, and has previously only been explored in transcriptomics studies (Reardon et al. 2021). Lastly, whilst the use of the 1st rank-ordered putatively annotated feature could provide biological insights (e.g., by linking it to a known MoA or AOP), the approach provides a less sensitive PODs due to the challenge of metabolite annotation in this study. To study the effect of sampling time points, the 1st rank-ordered unannotated feature was employed, which demonstrated a clear temporal trend for the aflatoxin B_1_ dataset: BMC values consistently decreased as exposure time increased up to 48 h, for all three nESI-DIMS assays measuring the polar metabolome and lipidome. However, this was not the case for HepaRG exposed to cyclosporin A, where the temporal trend was less apparent. For this dataset, it was possible to place a derived POD into an AOP framework for steatosis, as one of the putatively annotated features corresponded to a key event, the accumulation of triglycerides at 48 h. Such mechanistic anchoring highlights one of the applications of the AOP programme, although a metabolomics dataset must contain robust annotations in order for this approach to be reliable for regulatory purposes. As a result, the HepaRG-specific library of polar metabolites and lipids was prepared to aid these efforts. In summary, technological advances now enable large-scale metabolomics case studies of BMC modelling, although further work is required to improve the identification of metabolites and lipids, as well as their roles as key events in AOPs, in order for this approach to demonstrate value to regulatory toxicology.

## Supplementary Information

Below is the link to the electronic supplementary material.

Supplementary file1 (DOCX 610 KB)

Supplementary file2 (XLSX 76 KB)

## Data Availability

The datasets from the current study are available from the corresponding author upon request.
