# Supplementary material for: Derivation of metabolic point of departure using high-throughput in vitro metabolomics: investigating the importance of sampling time points on benchmark concentration values in the HepaRG cell line
Source: Arch Toxicol. 2023 Jan 22;97(3):721–35. doi: 10.1007/s00204-022-03439-3 (PMC9968698; doi:10.1007/s00204-022-03439-3)
Supplement: Supplementary file 1 — Supplementary file1 (DOCX 610 KB) [file 204_2022_3439_MOESM1_ESM.docx]

**Supplementary Information**

**Derivation of metabolic point of departure using high-throughput in vitro metabolomics: investigating the importance of sampling time points on benchmark concentration values in the HepaRG cell line**

Julia M. Malinowska ^1^, Taina Palosaari ^2^, Jukka Sund ^2^, Donatella Carpi ^2^, Ralf J. M. Weber ^1,3^, Gavin R. Lloyd ^3^, Maurice Whelan ^2^ and Mark R. Viant ^1,3,*^

^1^ School of Biosciences, University of Birmingham, Birmingham B15 2TT, UK

^2^ European Commission, Joint Research Centre (JRC), Ispra, Italy

^3^ Phenome Centre Birmingham, University of Birmingham, Birmingham B15 2TT, UK

* Correspondence: Mark R. Viant ([m.viant@bham.ac.uk](mailto:m.viant@bham.ac.uk))

**Supplementary Methods**

*HepaRG-specific library of polar metabolites and lipids*

Analysis of polar metabolites using HILIC UHPLC-MS/MS

HILIC analyses were conducted using an Accucore 150 Amide HILIC column (2.1 x 100 mm, particle size 2.6 μm, Thermo Fisher Scientific) with a column guard Accucore 150 Amide HILIC (2.1 x 10 mm, particle size 2.6 μm, Thermo Fisher Scientific) maintained at 35 °C in positive and negative ionisation modes as previously published (D’Elia et al. 2019; Southam et al. 2020, 2021). For positive ionisation mode, mobile phase A was 10 mM ammonium formate 95% acetonitrile:water with 0.1% formic acid (v/v), whilst mobile phase B was 10 mM ammonium formate 50% acetonitrile:water with 0.1% formic acid (v/v). For negative ionisation mode, mobile phase A was 10 mM ammonium acetate 95% acetonitrile:water with 0.1% acetic acid (v/v), whilst mobile phase B was 10 mM ammonium acetate 50% acetonitrile:water with 0.1% acetic acid (v/v). Ammonium formate (≥99%, LC-MS grade) and acetic acid (≥99.7%, LC-MS grade) were obtained from Fisher Chemical, whilst ammonium acetate (≥99.99% trace metals basis) and formic acid (~98%) were purchased from Honeywell. The chromatographic flow rate was 0.50 ml min^-1^, method duration was 15 min with the gradient applied as follows: t=0, 1% B; t=2.1, 1% B; t=4.1, 15% B; t=7.1, 50% B; t=10.1, 95% B; t=11.0, 95% B; t=11.5, 1% B; t=15, 1% B; with a curve of 5 (for both ionisation modes). The injection volume was 2 μL.

Mass spectrometry data was collected over *m*/*z* range 70-1050 with the following ion source parameters: spray voltage was either 3200 V (positive ionisation mode) or 2700 V (negative ionisation mode); sheath, auxiliary and sweep gases were set to 40, 8 and 1 arbitrary units, respectively; temperature of the ion transfer tube was 200 °C, whilst vapouriser temperature was 300 °C; radio frequency (RF) lens voltage was set to 35%. The sequence was composed of two injections of an extraction blank collected in full scan mode (with the second injection used as an exclusion reference), and two injections of polar metabolite extracts also collected in full scan mode (with the second injection used as an inclusion reference). Next, samples of polar metabolite extracts were analysed by collecting MS^n^ fragmentation data with seven ‘deep scans’ (i.e., iterative injections). This was followed by two injections of a solvent blank in a full scan mode. Within the sequence, the exclusion override factor was set to 3, exclusion duration was set to 10 s, and the selected preferred ions were [M+H]^+^ and [M-H]^-^. Two UHPLC-MS/MS datasets were collected; one including only MS^2^ fragmentation data, and the other one including MS^2^ and MS^3^ fragmentation (this was due to the earlier versions of the software used for data processing and analysis, Compound Discoverer by Thermo Scientific, being unable to process MS^3^ fragmentation data, a feature included in the release of Compound Discoverer version 3.2). Data in full scan mode was acquired using an Orbitrap mass analyser with a resolution of 120,000 (at *m*/*z* 200 full width at half maximum (FWHM)) and an AGC target of 4.0e5. Fragmentation data were collected using an Orbitrap mass analyser (MS^2^; resolution 30,000 (at *m*/*z* 200 FWHM); automatic gain control (AGC) target of 5.0e4) and an ion trap (MS^3^; rapid scan rate; AGC target of 1.0e4) with scan filters; exact parameters are included in Figure S1.

Analysis of lipids using RP C_30_ UHPLC-MS/MS

RP C_30_ analyses were conducted using an Accucore C_30_ column (2.1 x 150 mm, particle size 2.6 μm, Thermo Scientific) maintained at 55 °C in positive and negative ionisation modes as previously published (Jankevics et al. 2021). For positive and negative ionisation modes, mobile phase A was 20 mM ammonium formate 60:40 acetonitrile water (v/v), whilst mobile phase B was 20 mM ammonium formate 85.5:9.5:5 propan-2-ol:acetonitrile:water (v/v/v). The flow rate was 0.40 ml min^-1^, method duration was 30 min with the gradient applied as follows: t=0, 20% B; t=2.5, 20% B; t=2.6, 55% B; t=12, 60% B; t=12.1, 80% B; t=19, 90% B; t=21, 100% B; t=23, 100% B; t=23.1, 20% B, t=30, 20% B, with a curve of 5 (for both ionisation modes). The injection volume was 3 μL.

Mass spectrometry data was collected over an *m*/*z* range 150-2000 with the following ion source parameters: spray voltage was either 3200 V (positive ionisation mode) or 2600 V (negative ionisation mode); sheath, auxiliary and sweep gases were set to 40, 8 and 1 arbitrary units, respectively; temperature of the ion transfer tube was 300 °C, whilst vapouriser temperature was 350 °C; RF lens voltage was set to 70%. The sequence was composed as described for the HILIC analyses. The parameters employed for the collection of MS^2^ and MS^3^ fragmentation data in positive ionisation mode were based upon the methods described previously (Kiyonami et al. 2016; Peake et al. 2019) with additional mass and loss ‘triggers’ allowing for further characterisation of phosphatidylcholines and triglycerides (Figure S2). The parameters for the collection of MS^2^ fragmentation only in negative ionisation mode was as described for the HILIC analyses above, except for the duration of dynamic exclusion, which was set to 5 s.

**Fig. S1** Parameters employed for collecting MS^2^ and MS^3^ fragmentation data for the analysis of polar extracts using HILIC for chromatographic separation with an Orbitrap ID-X Tribrid mass spectrometer and AcquireX intelligent data acquisition workflow, with a cycle time of 0.6 s (Thermo Fisher Scientific).

**Fig. S2** Parameters employed for collecting MS^2^ and MS^3^ fragmentation data of lipids extracted from HepaRG hepatocytes using RP C_30_ chromatographic separation with an Orbitrap ID-X Tribrid mass spectrometer and AcquireX intelligent data acquisition workflow, with a cycle time of 1.5 s (for positive ionisation mode only) (Thermo Scientific).

Data processing and analysis of polar metabolites analysed by HILIC UHPLC-MS/MS

Data collected using HILIC UHPLC-MS/MS were processed and analysed using Compound Discoverer (Thermo Scientific, version 3.2.0.421). The workflow for data processing was composed of nodes and is described next.

1. Select spectra (default settings);
2. Align retention times (alignment model: adaptive curve, alignment fall back: use linear model, maximum shift (min): 0.1, shift reference file: true, mass tolerance: 5 ppm, remove outliers: true);
3. Detect compounds (mass tolerance: 5 ppm, intensity tolerance (%): 20, signal-to-noise threshold: 3, minimum peak intensity: 1e4, ion definitions: all available ion definitions except for [M-H+TFA]^-^ (32/33), base ions: [M+H]^+^, [M+NH4]^+^, [M-H]^-^, filter peaks: true, maximum peak width (min): 0.8, remove singlets: true, minimum number of scans per peak: 5, minimum number of isotopes: 1);
4. Group compounds (mass tolerance: 5 ppm, retention time tolerance (min): 0.1, preferred ions for fragment data selection: [M+H]^+^, [M+NH4]^+^, [M-H]^-^);
5. Mark background compounds (maximum ratio of sample to blank: 10);
6. Assign compound annotations (mass tolerance: 5 ppm, data sources: mzCloud Search (#1), Predicted Compositions (#2), ChemSpider Search (#3), Metabolika Search (#4), use mzLogic: true, use spectral distance: true, minimum spectral fit value: 20, maximum difference between fit value of best and worst candidates: 20);
7. Other annotations nodes included (with mass tolerance of 5 ppm for all nodes): mapping to Metabolika and Kyoto Encyclopedia of Genes and Genomes (KEGG) pathways, mzVault (using two available libraries contributed by Prof Bamba of Kyushu University), ChemSpider (databases: HMDB, KEGG, LIPID MAPS, search mode: by formula only), predict compositions (pattern matching included intensity tolerance and threshold of 20 and 0.1, respectively), search mzCloud (identity search: HighChem HighRes, match activation energy: any, activation energy tolerance: 20, match factor threshold: 60);
8. Apply mzLogic (FT fragment mass tolerance: 10 ppm, ion trap (IT) fragment mass: 0.4 Da, match factor threshold: 30);
9. Apply spectral distance (mass tolerance: 5 ppm, intensity tolerance (%): 20, intensity threshold: 0.1)

The putative polar metabolites detected in the HepaRG cell line were then filtered further; only metabolites with chromatographic retention times longer than 1 min, mass tolerance ± 5 ppm and a match score ≥60 in the mzCloud database were retained. Next, only polar metabolites present in at least 2 out of 3 technical replicates were kept, and their fragmentation data manually assessed. For some polar metabolites, the mzCloud match score was high (≥60) despite only having the parent ion for a match (thus the match was only based using accurate *m*/*z*); the confidence in these annotations was low and they were removed from the putative polar metabolite list. In addition, if a match was made only to an exogenous compound (according to the HMDB), it was removed from the final list. The metabolites present in the final list of polar metabolites detected in the HepaRG cell line (using HILIC UHPLC-MS/MS) were assigned (1) proposed putative names (for metabolites with L- and D-isomers, the more likely isomer was assigned (e.g., the L-isomer for amino acids), (2) HMDB identifiers (ID), (3) subclass according to HMDB, and (4) molecular formula. Furthermore, presence of polar metabolites in the MTox700+ metabolite panel was based on matching the HMDB IDs.

Data processing and analysis of lipids analysed by RP C_30_ UHPLC-MS/MS

Data collected using the RP C_30_ assay (i.e., fragmentation data obtained using the ‘deep scan’ workflow) were processed and analysed using LipidSearch (Thermo Scientific, version 4.2.29). The software was configured so that the auto validation after alignment process was enabled with the following settings: signal-to-noise ratio: 5, threshold of data points: 5, intensity ratio threshold: 3, intensity ratio baseline: 0.05. Given that only fragmentation data were processed in the LipidSearch, no blank subtraction was applied prior to this step. This was due to the precursor features present in the extraction blanks being automatically placed on the exclusion list by the AcquireX software, thus they were excluded from fragmentation. The workflow for data processing is described next. The target database was HCD with the option of the product search (mass tolerance of precursor and product ions: 5 ppm, intensity threshold for product ion: 1% (relative), and m-score threshold: 2.0) with the isotope correction algorithm correction on (‘recalc isotope’). Filters applied included (1) top-rank filter, (2) main isomer peak, (3) m-score threshold (display): 5.0, (4) fatty acid priority: on, and (5) quality filter A-D (with ‘A’ being of highest quality entailing complete identification of fatty acid chains and class (Kiyonami et al. 2016)). All lipid classes were selected for search with the following ion formations [M+H]^+^, [M+NH4]^+^, [M+Na]^+^, [M+K]^+^, [M+H-H2O]^+^, [M+2H]^2+^ for positive ionisation mode, and [M-H]^-^, [M+HCOO]^-^, [M-2H]^2-^, [M-CH3]^-^ for negative ionisation mode. Next, lipids were aligned across three technical replicates (each replicate being composed of seven iterative injections containing fragmentation data). The search and experiment type were set to ‘product’ and ‘LC-MS’, respectively, using the ‘maximum’ as an alignment method integration with the retention time tolerance of 0.15 min and calculation of unassigned peak areas on. Filters used included (1) the top rank filter on and (2) main node filter set to ‘all isomer peaks’, with (3) an mScore threshold of 5.0, and (4) quality (grade) filter A-D. Lipids were then filtered further manually: only lipids with the quality score of ‘BB-’ or higher were retained, entailing that (1) a compound had to be present in at least two out of three technical replicates and (2) the ‘ID quality’ according to LipidSearch was at least equal to ‘B’ in those two technical replicates corresponding to a lipid with an assigned class and some fatty acid chains (Kiyonami et al. 2016). The LipidSearch software assigned a compound with information including (1) lipid molecule, (2) class, (3) group, and (4) molecular formula. Lipids present in the final list of lipids detected in the HepaRG cell (using the RP C_30_ assay) were manually searched against the LIPID MAPS database using the information obtained from the LipidSearch software (i.e., molecular formula and lipid class). In addition, presence of the lipids in the MTox700+ metabolite panel was based on matching the HMDB IDs. Given that the *sn-*position could not be assigned, all possible available combinations were included in the information retrieved from the LIPID MAPS database.

**Supplementary Results**

**Table S1**

mRSD (%) of spectral feature intensities for the intrastudy QC sample (repeatedly measured throughout each assay) and the negative control samples (i.e., cells in media containing 0.1% DMSO), at each sampling time point for the three assays. Each dataset corresponds to the processed peak intensity matrix immediately prior to the univariate data analysis.

| **Assay** | **Feature count** | | | **mRSD (%): intrastudy QCs** | **mRSD (%): negative control samples per sampling time point** | | | | |
| --- | --- | --- | --- | --- | --- | --- | --- | --- | --- |
|  | |  |  | | **2 h** | **6 h** | **12 h** | **24 h** | **48 h** |
| Polar positive | | 3053 | 13.9 | | 28.7 | 27.2 | 26 | 25.1 | 29 |
| Polar negative | | 4678 | 12.3 | | 22.6 | 27.1 | 22.9 | 23.4 | 22.7 |
| Lipid positive | | 3274 | 13.1 | | 24.2 | 25.4 | 26.8 | 24.2 | 21.4 |


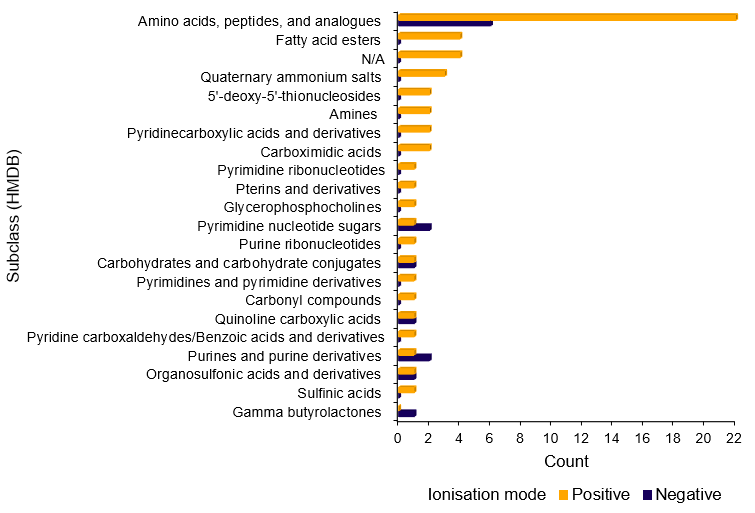


**Fig. S3** Subclasses of polar metabolites present in a curated library of expected metabolites of the HepaRG cell line, measured by HILIC UHPLC-MS/MS in positive and negative ionisation modes. The subclass of a metabolite was assigned according to the HMDB (version 4.0).


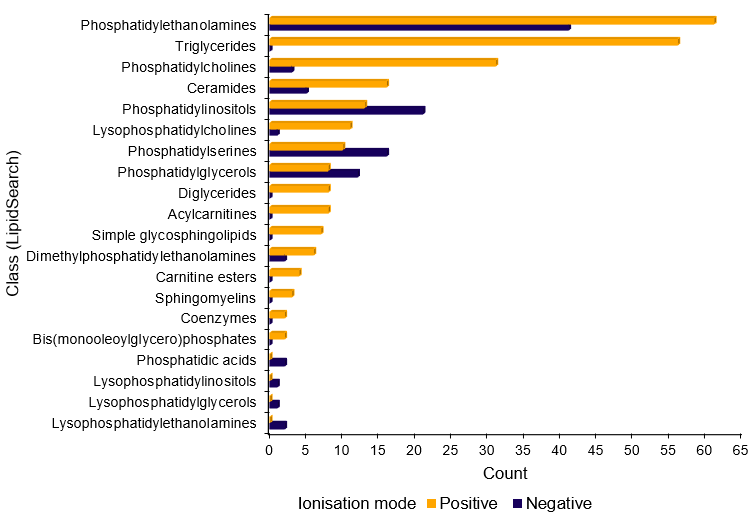


**Fig. S4** Classes of lipids present in a curated library of expected metabolites of the HepaRG cell line, measured by the RP C_30_ UHPLC-MS/MS assay in positive and negative ionisation modes

. The class of a lipid was obtained through LipidSearch (version 4.2.29).

**
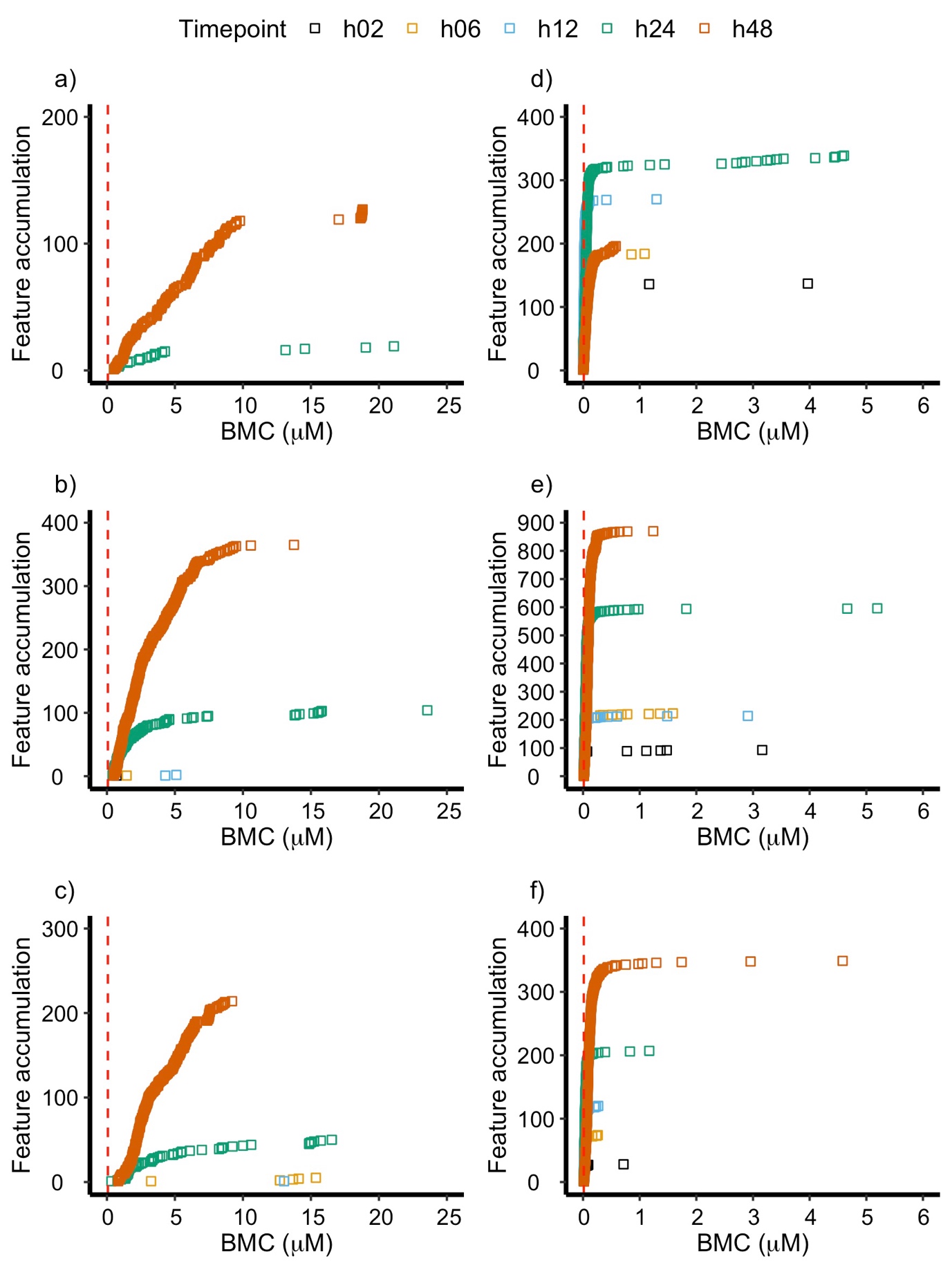
**

**Fig. S5** Accumulation plots corresponding to the exposure of the HepaRG cell line to benzo[a]pyrene (a-c) and rotenone (d-f) at five sampling time points. The metabolome was measured by polar positive (a,d), polar negative (b,e) and lipid positive (c,f) nESI-DIMS assays. The plot demonstrates accumulation of features with concentration-response behaviour ordered by the lowest BMC value. The red dashed line indicates the lower limit of extrapolation as suggested by the National Toxicology Program (i.e., 1/3 of the lowest experimental concentration in the study) (Auerbach et al. 2018).


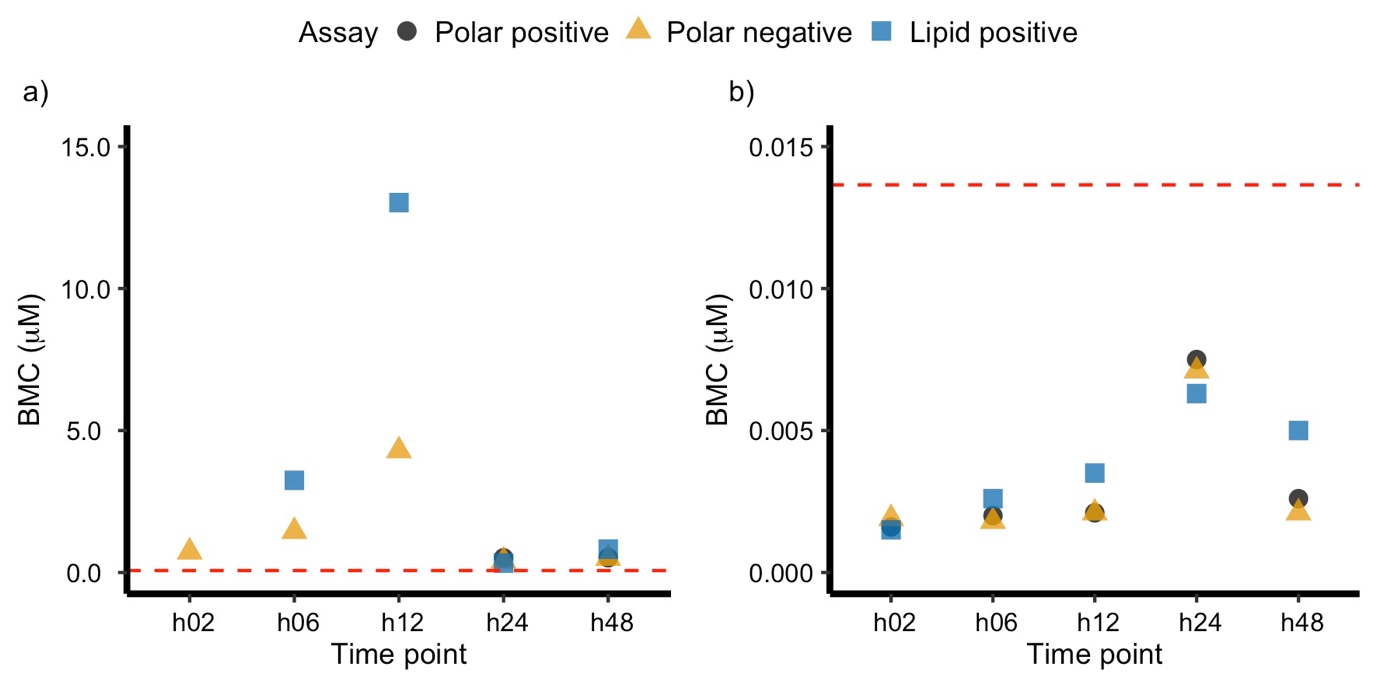


**Fig. S6** Plots demonstrating the effect of exposure time on BMC values derived from the metabolic responses of the HepaRG cell line exposed to a) benzo[a]pyrene, and b) rotenone, obtained by three nESI-DIMS assays measuring the polar metabolome and lipidome. The approach used to derive the BMC value for each time point and assay corresponds to the 1^st^ rank-ordered unannotated feature. The red dashed line indicates the lower limit of extrapolation as suggested by the National Toxicology Program (i.e., 1/3 of the lowest experimental concentration in the study) (Auerbach et al. 2018).

**References**

Auerbach S, Behl M, Collins B, et al (2018) NTP Research Report on In Vivo Repeat Dose Biological Potency Study of Triphenyl Phosphate (CAS No. 115-86-6) in Male Sprague Dawley Rats (Hsd: Sprague Dawley SD) (Gavage Studies)

D’Elia RV, Goodchild SA, Winder CL, et al (2019) Multiple metabolic pathways are predictive of ricin intoxication in a rat model. Metabolomics 15:1–15. https://doi.org/10.1007/s11306-019-1547-9

Jankevics A, Jenkins A, Dunn WB, Najdekr L (2021) An improved strategy for analysis of lipid molecules utilising a reversed phase C30 UHPLC column and scheduled MS/MS acquisition. Talanta 229:. https://doi.org/10.1016/j.talanta.2021.122262

Kiyonami R, Peake DA, Yokoi Y, Miller K (2016) Increased Throughput and Confidence for Lipidomics Profiling Using Comprehensive HCD MS 2 and CID MS 2 / MS 3 on a Tribrid Orbitrap Mass Spectrometer. Thermo Fisher Application Note 648:

Peake D, Kiyonami R, Yokoi Y, et al (2019) Increased confidence of insect lipidome annotation from high-resolution Orbitrap LC/MSn analysis and LipidSearch software. Thermo Fisher Scientific, Application Note 72942

Southam AD, Haglington LD, Najdekr L, et al (2020) Assessment of human plasma and urine sample preparation for reproducible and high-throughput UHPLC-MS clinical metabolic phenotyping. Analyst 145:6511–6523. https://doi.org/10.1039/d0an01319f

Southam AD, Pursell H, Frigerio G, et al (2021) Characterization of Monophasic Solvent-Based Tissue Extractions for the Detection of Polar Metabolites and Lipids Applying Ultrahigh-Performance Liquid Chromatography-Mass Spectrometry Clinical Metabolic Phenotyping Assays. Journal of Proteome Research 20:831–840. https://doi.org/10.1021/acs.jproteome.0c00660
